# The Effects of Physical Therapy in the Rehabilitation of Motor Delays in Children with Down Syndrome: A Systematic Review

**DOI:** 10.3390/jcm15051717

**Published:** 2026-02-24

**Authors:** Dan Alexandru Szabo, Adina Stoian, Cristina Veres, Heidrun Adumitrachioaie, Carmen Pârvu, Ioan Teodor Hășmășan, Ioan Sabin Sopa

**Affiliations:** 1 Department M2, Faculty of Medicine, George Emil Palade University of Medicine, Pharmacy, Science, and Technology of Targu Mures, Gheorghe Marinescu Street 38, 540139 Targu Mures, Romania; dan-alexandru.szabo@umfst.ro; 2 Department of Pathophysiology, George Emil Palade University of Medicine, Pharmacy, Science and Technology of Targu Mures, 540139 Targu Mures, Romania; adina.stoian@umfst.ro; 3 Department of Industrial Engineering and Management, George Emil Palade University of Medicine, Pharmacy, Science, and Technology of Targu Mures, Nicolae Iorga Street 1, 540088 Targu Mures, Romania; 4 Department of Pediatrics, George Emil Palade University of Medicine, Pharmacy, Science, and Technology of Targu Mures, Gh. Marinescu Street, 38, 540136 Targu Mures, Romania; 5 Department for Teacher Training, Faculty of Educational Sciences, “Dunărea de Jos” University, Științei Street 2, Block G, 800146 Galati, Romania; 6 Department of Environmental Sciences, Physics, Physical Education and Sports, “Lucian Blaga” University Sibiu, 550012 Sibiu, Romania; ioan.hasmasan@ulbsibiu.ro (I.T.H.); sabin.sopa@ulbsibiu.ro (I.S.S.)

**Keywords:** physical therapy, rehabilitation, motor delays, Down syndrome

## Abstract

**Background/Objectives**: This study’s primary goals are to evaluate the effects of physical therapy on motor delays in children with Down Syndrome, identify the most successful interventions, look at current trends in the field, and suggest future directions for clinical and research development by reviewing the scientific literature published over the past ten years. **Methods**: Using reputable databases, including PubMed, ScienceDirect, CENTRAL (Cochrane Central Register of Controlled Trials), PEDro (Physiotherapy Evidence Database), Web of Science, and NIH, an electronic search of scholarly literature was carried out between January and April 2025. To organise the findings and select the most pertinent papers, a search strategy was required. **Results**: The studies analysed provide a complex picture of how different types of physical therapy interventions affect children and adolescents with Down syndrome. **Conclusions**: Physical therapy interventions suggest greater effectiveness during the early stages of motor development in children with Down Syndrome; however, the evidence, based on six heterogeneous studies, remains moderate and does not support definitive recommendations. In clinical practice, physical therapists are advised to design individualised programmes that address specific needs, utilising traditional therapies, online training, or movement stimulation techniques, and to systematically monitor their outcomes.

## 1. Introduction

From birth, every child grows and learns at their own pace. However, certain skills are expected to be achieved at specific age-related milestones. These can be physical, language-related, or social skills [1,2,3,4].

The ability to move is essential for human life and development. From birth, children develop a variety of motor skills. Gross motor skills involve large-scale movements of the whole body and are necessary for performing functional activities [5,6]. These skills are fundamental not only for play and physical activities but also for acquiring competence in self-dressing, developing strength and endurance, and participating in tasks requiring fine motor skills [7,8].

In paediatric rehabilitation (PR), physical therapy (PT) plays an important role; however, it is not an isolated process. Instead, it must be part of an interdisciplinary treatment programme in which collaboration among specialists, such as occupational therapists, nutritionists, speech therapists, and psychotherapists, is vital to achieving optimal outcomes [9,10].

Paediatric PT uses neuromotor development techniques to help children with neurodevelopmental disorders improve their motor control, postural alignment, and functional movement [11]. These approaches are based on an understanding of the processes of children’s movement learning and development. This enables therapeutic interventions to elicit optimal movement patterns. An example of a neuromotor development technique is the Bobath method. This therapy is a complex, personalised approach to treating patients with neurological disorders, emphasising the re-education of normal movement and the improvement of functional outcomes. It is based on manually facilitating appropriate movements and integrating them into everyday life, thereby helping patients regain motor control and independence [12].

Down Syndrome (DS) is an increasingly common diagnosis both in Europe and worldwide, and the issue of motor delays in children with this syndrome is a topic of significant relevance in the context of paediatrics and neurophysiotherapy due to the need for specialised interventions that these patients require [13,14]. Due to significant advances in medicine, people with DS have a much longer life expectancy, which makes the inclusion of therapies in their recovery programme increasingly important, as it influences not only their longevity but also their quality of life [15,16].

People with DS can acquire the same basic motor skills necessary for daily life and personal independence. Although children with this syndrome develop motor skills in roughly the same order as children without this diagnosis, these skills are typically achieved later and with lower precision [17,18].

The motivation for this research stems from the need to address knowledge gaps that would enhance the efficacy of PT in managing motor delays specific to DS. This condition is characterised by hypotonia, joint instability, and developmental delays, which require a personalised approach.

The primary objective of this article is to analyse and highlight the impact of integrating PT on motor development in children with DS, with an emphasis on improving motor function, posture, balance, and functional independence. The study’s second objective is to highlight the role of PT interventions in reducing the motor delays characteristic of this population. The third objective of this paper is to highlight the importance of early intervention and to establish evidence-based best-practice guidelines to support professionals and families throughout the PR process.

By analysing the specialised literature, the paper will examine whether these interventions significantly improve motor development, posture, and function, providing evidence to support their use in clinical practice.

The structure of this paper is as follows: In Section 2, the Study search strategy, Inclusion Criteria, Exclusion Criteria, and Methodological Quality are presented. In Section 3, Assessment of Methodological Quality, the Methodological Quality Analysis, and the Summary of Results are presented.

Finally, Section 4 discusses the study’s limitations and future research directions, and Section 5 presents the Conclusions. Appendix A.1 contains the PEDro Scale, and Appendix A.2 encompasses the Strobe statement.

## 2. Materials and Methods

Our study is a systematic review of heterogeneous designs that intentionally integrate multiple types of evidence using assessment and synthesis methods appropriate to the project, and then integrate them transparently so that the conclusions reflect the distinct contributions.

**Figure 1:**
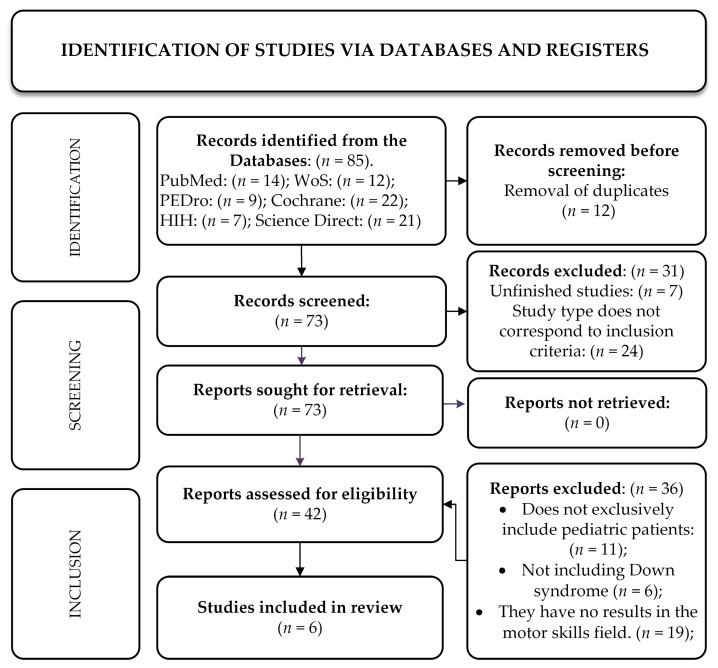
Study identification in accordance with the PRISMA reporting guidelines (PRISMA 2020).

This systematic review, conducted in accordance with the PICOS methodology [19] and PRISMA reporting guideline (PRISMA 2020) (Figure 1), aims to answer the following research question: In children with Down syndrome, do PT interventions, compared with no specific intervention or standard care, improve motor development?

Consequently, in this paper, we review the scientific literature published over the last ten years to assess the effects of PT on motor delays in this paediatric population, identify the most effective interventions, examine current trends in the field, and outline potential directions for clinical and research development. Research manuscripts reporting larger datasets deposited in a publicly available database should specify the database and provide the relevant accession numbers. If the accession numbers are not yet available at the time of submission, please indicate that they will be provided during review. They must be provided prior to publication.

### 2.1. Study Search Strategy

Between January and April 2025, an electronic search of scientific literature was conducted using reliable databases, namely: PubMed, ScienceDirect, CENTRAL (Cochrane Central Register of Controlled Trials), PEDro (Physiotherapy Evidence Database), Web of Science, and NIH. A search strategy was necessary to organise the results and select the most relevant studies.

For this systematic review, only studies that suggested high methodological rigour and clinical relevance were included. Thus, the following types of studies were selected: randomised controlled trials, controlled clinical trials, pre-post-study studies, pilot studies, prospective-retrospective studies, and relevant case studies. These studies were selected for their ability to provide precise data on the effectiveness of PT interventions for children with DS.

Below is a table (Table 1) illustrating the basic data and keywords used for each database, along with the Boolean operators applied.

### 2.2. Inclusion Criteria

The standards used to determine which studies to include in the review were based on the PICOS research strategy [19] (Population, Intervention, Comparison, Outcomes, Study) (Table 2). This decision was made to provide a clear answer to the research question about the effectiveness of physiotherapy interventions for children with DS on motor development. The studies included in the research were those that included groups of children with DS who received PT interventions, such as the Bobath, Vojta, Cuevas Medek methods, and structured exercise programmes. The comparison group consisted of children who did not receive any intervention or were compared with another type of therapy, provided that both types fell within the chosen methods, and the results were evaluated for both groups. In some studies, children with typical development served as the control group. The results focused on motor development, which was assessed using validated instruments.

### 2.3. Exclusion Criteria

The following studies were excluded:

- Those that did not exclusively include children with DS aged 0 to 18 years.
- Studies that focused on outcomes other than motor development.
- Studies that did not directly assess the impact of PT or physiotherapy interventions on motor development.

In addition, for reasons of the evidence’s relevance and timeliness, studies that were not available in full text, review articles, editorials, or isolated case studies, as well as those published before 2014, were excluded.

### 2.4. The Methodological Quality

The PEDro scale [20] (Appendix A.1), a specialised tool for evaluating physiotherapy clinical trial methodology, was used to assess the methodological quality of the randomised controlled trials included in the study. The PEDro scale [20] is a valuable resource for supporting evidence-based clinical practice in experimental studies. This scale includes criteria for external validity (criterion 1), internal validity (criteria 2–9), and statistical information for interpreting clinical trial results (criteria 10 and 11). Finally, studies are classified into low quality (scores below 4), moderate (scores 4–5), good (scores 6–8), and exceptional (scores 9–10).

To assess the methodological quality of the observational studies included in this systematic review, a tool known as the STROBE scale [21] (Appendix A.2) (Strengthening the Reporting of Observational Studies in Epidemiology) was used. This tool comprises 22 criteria that address essential elements of studies, including study design, participant selection, statistical methods, and interpretation of results.

The study complied with the ethical principles described in the Declaration of Helsinki (1964) and its amendments, as adopted by the 75th General Assembly of the World Medical Association (WMA) in October 2024, Helsinki, Finland. Furthermore, the development and reporting of this systematic review followed the PRISMA (Preferred Reporting Items for Systematic Reviews and Meta-Analyses) guidelines. Given that the two methodological assessment scales, PEDro and STROBE, were included in the study, the protocol did not require registration in PROSPERO.

## 3. Results

Between January and April 2025, an electronic search of scientific literature was conducted using reliable databases, namely: PubMed, ScienceDirect, CENTRAL (Cochrane Central Register of Controlled Trials), PEDro (Physiotherapy Evidence Database), Web of Science, and NIH.

The selection of studies for this systematic review was conducted in accordance with the PICOS criteria and the defined inclusion and exclusion criteria. Using the aforementioned databases and Boolean operators, 85 studies were identified, of which six met the inclusion criteria for the review.

Titles and abstracts were independently reviewed by two evaluators (co-authors), who subsequently conducted independent full-text assessments. Data extraction was conducted in duplicate using an Excel file. Any discrepancies were initially resolved through discussion and consensus, with unresolved disagreements adjudicated by a third evaluator (co-author). Study selection and data extraction processes were documented in a Word file.

The process by which the studies for this systematic review were selected is presented in the flow chart below.

### 3.1. Assessment of Methodological Quality

The six studies included in the systematic review were published between 2014 and 2025 and are as follows:

- “Effect of Structured Exercise Program on Fundamental Motor Skills in Children with Down Syndrome: Pilot study”, written by Myo Thein Tun et al., 2021 [22].
- “Comparison of the effectiveness of Bobath and Vojta techniques in babies with Down syndrome: Randomized controlled study”, written by Erdogan Kavlak et al., 2021 [23].
- “Early physiotherapy and Down Syndrome: does this improve aEG of walking?” Written by H. Towse et al., 2016 [24].
- “Effects of early physical therapy on motor development in children with Down syndrome,” written by Feyzullah Necati Arslan et al., 2022 [25].
- “Influence of additional weight on the frequency of kicks in infants with Down syndrome and infants with typical development” written by Gabriella L. Santos et al., 2014 [26].
- “A Tele-Coaching Pilot Study: An Innovative Approach to Enhance Motor Skills in Adolescents With Down Syndrome” written by Matteo G. et al., 2025 [27].

All studies focus mainly on the effect of PT on motor skills. The patients included in these studies are aged 0–18 years and have been diagnosed with DS. A total of 142 patients were included in these studies, of whom five had typical development and were not diagnosed with DS or any other disease; they comprised the control group in the study by Santos et al. [26]. In Table 3, we present all key characteristics of the study.

In three studies, namely those written by Gabriella L. Santos et al. [26], H. Towse et al. [24], and Feyzullah N.A. et al. [25], we have control groups, and in the latter two, both the experimental and control groups are diagnosed with DS. The other studies analyse the results of therapy before and after its application on a single group.

In five of the six studies, the treatment focuses on PT exercises to improve gross and fine motor skills, coordination, balance, and gait, and is delivered in weekly sessions. The last study [27] focuses on a precursor movement to walking, namely the lower-limb movements of infant patients, which are necessary for normal development, and it includes two sessions, at 3 and 4 months of age.

### 3.2. Methodological Quality Analysis

Two standardised instruments were used to assess the methodological quality of the studies included in this systematic review: the PEDro scale [20] and the STROBE checklist (Table 4) [21]. These scales were used to enable an impartial assessment of the internal and external validity, methodological rigour, and reporting quality of each study. Table 5 shows the scores obtained by each study. This helps us better understand the extent to which we can be confident in the study results.

### 3.3. Summary of Results

The studies analysed provide a complex picture of how different types of PT interventions affect children and adolescents with Down syndrome.

A structured three-week exercise programme was implemented to improve fundamental motor skills and lower-limb functional strength. This was demonstrated by Myo Thein Tun et al. [22]. However, the intervention did not have a statistically significant effect on static balance. This indicates that some aspects of motor control require longer interventions to achieve noticeable progress.

The motor performance of infants with DS was significantly improved by both Bobath and Vojta therapies, according to the results reported by Kavlak E. et al. [23]. Both approaches were shown to be effective, with no significant differences between groups, suggesting that both are suitable alternatives for early intervention. In contrast, a study by Towse H. et al. [24] showed that children with DS who received early PT did not achieve independent walking at an earlier age. In the control group, without physical therapy, a similar age of walking acquisition was observed, but these differences were not statistically significant.

Another study by Feyzullah N.A. et al. [25] showed that participation in frequent PT programmes, especially when therapy began before age one, had a significant positive effect on gross and fine motor skill development. The authors strongly support the importance of immediate intervention to maximise the motor potential of children with Down syndrome.

Examining the impact of added weight on stroke frequency in infants, Gabriella L. Santos et al. [26] addressed a less studied aspect. Both the typically developing and DS groups showed increased movement frequency and task success following the addition of weight.

The study by Matteo Giuriato et al. [27] showed that an internet-based exercise programme significantly improved balance and systolic blood pressure in adolescents but had no significant effect on adiposity. These findings support the addition of online interventions to community programmes that help people with Down syndrome.

The six included studies showed substantial heterogeneity in intervention type (e.g., task-specific training, neuromuscular stimulation, and parent-led programmes), duration, intensity, session frequency, participant age ranges, targeted outcomes, and measurement tools. This variability resulted in inconsistent comparators and outcome metrics, preventing meaningful synthesis of results and limiting the ability to determine a single most effective rehabilitation approach for motor delays in children with DS.

Studies with higher methodological quality, as assessed by the PEDro and STROBE scales (Towse et al., Feyzullah et al., Matteo G. et al. [24,25,27]), informed the synthesis by clarifying the weighting of different study designs. Findings from study designs with stronger levels of evidence and higher quality ratings were given greater influence in the overall interpretation. In contrast, lower-quality or single-case designs (Myo Thein Tun et al. [22]) were regarded as hypothesis-generating and interpreted with appropriate caution. Conclusions were primarily based on higher-quality evidence, notably controlled studies with superior methodological scores, which were weighted more heavily in our synthesis and interpretation of findings.

## 4. Discussion

The study’s purpose was achieved: to review the scientific literature published over the last 10 years to assess the effects of PT on motor delays in this paediatric population, identify the most effective interventions, examine current trends in the field, and outline potential directions for clinical and research development.

The Discussion section was designed to address knowledge gaps to improve PT’s effectiveness in managing DS-specific motor delays. All of these knowledge gaps are addressed here, from the perspective of each study reviewed.

According to most studies, motor development is characterised by significant improvements in fine and gross motor skills, balance, and lower-limb functional strength. In addition, early interventions, especially those initiated before age 1, have shown better outcomes, underscoring the importance of early treatment in addressing motor delays.

For children with Down syndrome, the studies included in this review showed a significant influence of PT interventions on motor delays. There were notable differences in the type of therapy applied and its duration. The study by Kavlak et al. (2022) [23], which scored 6/10 on the PEDro methodological quality assessment table, examined 23 children with Down syndrome. They were divided into two groups: one received Bobath treatment and the other Vojta treatment. After treatment, both groups showed significant improvements in motor development, as well as in mothers’ emotional state and quality of life, indicating that both methods are effective in reducing motor delays in children. In addition, the study by Feyzullah et al. (2022) [25], which had some SROBE biases and generalizability limitations, provided further evidence of PT’s benefits for motor development in children aged 6 to 42 months. The study found significant improvements in fine and gross motor skills with regular physical therapy, particularly among children who received it before age 1. This result underscores the importance of early interventions and supports the conclusion that they can reduce motor delays in children with DS [23,25].

In contrast, the study by Towse et al. (2016) [24], a study that presents some methodological/reporting deficiencies, indicating the absence of some STROBE elements (study design, context, quality of results, data sources) examined the impact of PT on age at walking in children and found no significant difference between children who received PT and those who did not. However, the author noted a significant difference in walking age between Caucasian and non-Caucasian children, suggesting that sociocultural factors and the availability of health services may influence outcomes. Despite this less conclusive result, the study confirms that physical therapy can reduce motor delays, although the effect was not statistically significant [24].

Gabriella S. et al. (2014) [26] conducted another relevant study, which scored 6/10 on the PEDro methodological quality assessment scale, examining the motor development of 10 children: 5 with DS and 5 with typical development. A mobile device was used in this study to measure infants’ spontaneous motor activity. These measurements included the frequency of kicks, contact with a touch pad, and the ability to lift it under several experimental conditions, including those in which the infants were subjected to additional weight. The results showed that infants with DS and those with typical development suggesting significant improvements in motor performance when subjected to additional weight, and that, when the weight was removed, they continued to perform the movement better. This result suggests that motor stimulation through early exercise, including weight-bearing activities, may contribute to motor development in children with DS and beyond [26].

In another example, Myo Thein Tun et al. (2021) [22], a study with methodological/reporting deficiencies, indicating the absence of the STROBE elements, biases, and generalizability and other general information, examined five children with DS and found that, after three weeks of intervention, fundamental motor skills, particularly balance and lower-limb functional strength, improved significantly. According to this study, even in a relatively short period, short-term interventions can significantly affect motor skills. In addition, a study by Matteo G. et al. (2025) [27], which does not include a STROBE checklist describing efforts to address potential sources of bias, reported significant improvements in balance and cardiovascular health among adolescents with DS after 15 weeks of online training. The results of this study showed that PT interventions can be beneficial across early development and adolescence [22,27].

Across all included studies, PT interventions were beneficial for children with DS in motor development, with significant improvements observed in most cases. Even with short-term interventions, studies by Myo Thein Tun et al. (2021) [22] and Matteo G. et al. (2025) [27] suggested benefits for fine and gross motor skills, balance, and functional strength. These interventions had a remarkable impact within a relatively short period, indicating that changes can be observed even in older children and adolescents, not only in the early years of life. However, notable differences exist among the included studies, particularly in the types and durations of the interventions. For example, the studies by Kavlak et al. (2022) [23] and Feyzullah et al. (2022) [25] used traditional therapies, such as Bobath and Vojta, which are well known in physiotherapy practices, while the studies by Matteo G. et al. (2025) [27], Myo Thein Tun et al. (2021) [22], and Gabriella S. et al. (2014) [26] used more modern methods, such as online training and short-term physiotherapy sessions. The treatment of motor delays in children with DS can be effective with a wide range of interventions, as suggested by these different methods. Another significant distinction between the studies was sample size and intervention duration. Small group studies, such as those by Myo Thein Tun et al. (2021) [22], had problems generalising their results, while studies with large groups, such as those by Kavlak et al. (2022) [23], had problems directly comparing treatment effects due to variations in the type and duration of interventions. In addition, the study by Towse et al. (2016) [24] examined access to PT and found differences between Caucasian and non-Caucasian groups, suggesting that socioeconomic and cultural factors may affect outcomes, which have not always been accounted for in other studies.

One advantage of the studies presented in this systematic review is the diversity of intervention types and populations, which enhances the research and improves understanding of the effects of PT on motor delays. For example, studies by Kavlak et al. (2022) 23] and Feyzullah et al. (2022) [25] obtained clear, comparable data using standardised assessments and valid measures, such as the Bayley III and the 6 min walk test. In addition, studies of children with DS from infancy through adolescence provide an in-depth picture of how PT interventions affect motor development across developmental stages. Despite this, there are also significant weaknesses. Retrospective studies, such as that by Towse et al. (2016) [24], may be subject to reporting biases. In addition, the studies by Myo Thein Tun et al. (2021) [22] and Matteo G. et al. (2025) [27] have small sample sizes, limiting the generalizability of their results and making it difficult to assess the long-term effects of interventions. To strengthen the conclusions, further studies with larger control groups and long-term follow-ups would be necessary.

The results of this systematic review are consistent with previous research suggesting the effectiveness of physical therapy interventions in improving motor development in children with DS. For example, Rodríguez-Grande et al. (2022) [28] conducted another systematic review evaluating the effects of therapeutic exercises (aerobic, resistance, neuromuscular, or neuromotor) in children with DS aged 0 to 3 years, a period during which therapeutic interventions are essential for promoting appropriate motor patterns. The study concluded that, compared with daily activities, exercise therapy is more effective at improving gait and motor development in children with Down syndrome. In addition, aerobic treadmill exercise, performed 5 days per week for 6 to 8 min at an intensity of 0.2 to 0.5 m per second, can significantly affect motor development. Therefore, both reviews support the conclusion that exercise therapies help children with DS develop appropriate motor behaviour, underscoring the importance of immediate and continuous interventions.

### 4.1. Limitations of the Study and Future Research Directions

Several limitations should be considered in this systematic review. One significant factor is the small number of included studies, which limits the generalizability of the conclusions and may reduce the external validity of the results. A broader selection of studies would have been beneficial for providing a more detailed assessment of the effectiveness of PT interventions and for identifying potentially significant differences among therapeutic methods. The comparison and interpretation of results were affected by variations in the assessment tools used across studies. There were also significant differences in the types of interventions and their durations, which adds another degree of variability to the assessment of therapeutic effects. The next limitation is that the included studies were predominantly published in English, potentially excluding relevant research in other languages. Another limitation of the study is that it includes small pilot studies, and some of the studies lack a control group, which may slightly affect the reliability of the conclusions. Although PEDro and STROBE scores are presented, their implications are not critically integrated into the discussion. These methodological shortcomings may have partially influenced the results, and the overall evidence quality is low to moderate; PEDro/STROBE results limit confidence in the pooled conclusions. The study limitations included the use of Boolean operators for keyword searches, restricting the publication period to 2015–2025, and limiting the studies to English to ensure complete understanding.

Finally, this study’s conclusions are limited by the small number of eligible studies, often small sample sizes, substantial heterogeneity in interventions and outcomes, variable methodological quality, potential publication bias favouring positive results, and language bias restricting evidence to certain languages, all of which substantially reduce confidence and generalizability of the findings.

Future studies should focus on randomised controlled trials with larger samples. This should be done to confirm that PT interventions help reduce motor delays and to find the most effective treatments. To evaluate the long-term effects of PT interventions on motor development in children with DS, it would also be beneficial to implement standardised treatment and progress monitoring protocols, including the use of digital technology.

### 4.2. Clinical Implications

It is important to emphasise that these studies provide a complex picture of how different types of PT interventions affect children and adolescents with Down syndrome, so that all this data can be used in clinical practice to provide a comprehensive picture of how different types of PT interventions affect children and adolescents with Down syndrome, in order to support the importance of immediate intervention to maximise the motor potential of children with Down syndrome. Finally, interdisciplinary and, in some cases, transdisciplinary collaboration is recommended to standardise patient education and improve understanding of the neurological mechanisms underlying motor delays, with the specific goal of optimising rehabilitation practice and providing clinically relevant insights for paediatric neurology.

## 5. Conclusions

This systematic review addressed the question: “Do PT interventions have significant effects on motor delays in children with DS?” The studies indicate that PT interventions significantly reduce motor delays in paediatric patients with DS. The results of this review, based on six heterogeneous studies, indicate that PT interventions can significantly reduce motor delays in children with DS, especially when implemented early. In practice, because interventions are most effective early on, physical therapists should pay particular attention to developing personalised treatment programmes tailored to each child’s individual needs. These interventions may include both traditional therapies, such as Bobath and Vojta, and online training or movement-stimulation techniques, selected according to each patient’s needs.

When investigating the rehabilitation of motor delays in children with DS, researchers should account for potential confounding factors, including socioeconomic status, cultural beliefs and practices, ethnicity, medical comorbidities, and varying access to rehabilitation services. These factors may independently influence motor development, parental involvement, and adherence to interventions, thereby affecting outcomes beyond the direct effects of PT and potentially impacting conclusions regarding treatment efficacy.

The review’s overall conclusions indicate that PT interventions may improve motor development, particularly when initiated early; however, the evidence remains limited and heterogeneous.

## Figures and Tables

**Table 1:** Databases and search criteria.

Databases	Search Terms
PubMed CENTRAL (Cochrane Central Register of Controlled Trials)	(“Down syndrome” OR “Trisomy 21”) AND (“motor delay” OR “motor impairment” OR “motor development” OR “gross motor skills”) AND (“physical therapy” OR “physiotherapy” OR”rehabilitation” OR “exercise therapy”) AND (“children” OR “pediatric” OR “infant” OR “adolescent” OR “young patients”) AND (“effect” OR “outcome” OR “improvement” OR “efficacy”)
PEDro	(“Down syndrome” AND “physical therapy”)
ScienceDirect	(Down syndrome) AND (“motor delay” AND “motor development” OR “gross motor skills”) AND (“physical therapy” OR “physiotherapy” OR “rehabilitation”) AND (“effect” OR “improvement”)
Web of Science	(“ Down syndrome”) OR (“ motor skills”) OR (“ physical therapy”) AND (“ children”) AND (“ effect”)
NIH	(“ Down syndrome/rehabilitation”) AND (“ physical therapy”) AND (“ children”) AND (“ motor development”) AND (“ effects”) AND (“ outcome”)

**Table 2:** PICOS Components.

Component	Description
**P**	Children diagnosed with Down syndrome
**I**	PT interventions, including Bobath, Vojta, Cuevas Medek, and physical exercises.
**C**	No intervention, standard care, or comparison with typically developing children
**O**	Motor development outcomes
**S**	Primary studies: RCTs, controlled clinical trials, pre-post, prospective and retrospective studies, relevant case studies, pilot studies

**Table 3:** Main characteristics of the included studies.

Author/Year/Type of Study	Sample Size (m/f)	The Intervention	Type of Therapy/Duration	Variables/Measuring Instruments	Results
Gabriella L. Santos et al. [26]. Quasi-experimental study	*n* = 10 EG:5 (3/2) CG: 5 (3/2) CG: typically developing children	For both groups: motivating kicks toward a target by stimulating the visual and auditory senses upon hitting it	For both groups: motivating kicks toward a target by stimulating the visual and auditory senses upon hitting it	1. Frequency of strikes 2. Frequency of strikes on target 3. Frequency of success in activating stim. Visual Measurements were taken before/during/after adding weights.	1. EG: *p* = 0.011 CG: *p* = 0.159 2. EG = CG: *p* = 0.013 3. EG: *p* = 0.001 CG: *p* = 0.679
Towse H. et al. [24]. Observational/retrospective study	*n* = 28 EG: 11 CG: 18	EG: Personalised kinesitherapy exercises CG: No therapy for motor development	EG: Kinesitherapy exercises at a qualified centre. Exercises to improve motor skills.	The age, in months, at which the patient began walking independently. Ethnicity	EG: 27.7 months CG: 22.6 months *p* = 0.08 No statistical difference between groups. *p* = 0.025 showing a statistical difference between ethnic groups (non-Caucasian group 19.5 months).
Feyzullah Necati Arslan et al. [25]. Observational study	*n* = 58 EG: 34 (21/13) CG:24 (9/15)	EG: specific physical therapy exercises to improve motor skills, at a specialised centre for at least 12 weeks CG: No therapy	EG: kinesitherapy exercises 2 days/week Approx. 45 min/session The programme is based on balance, muscle strength, and coordination exercises.	1. Bailey III 2. FMSS (fine motor skills scale—part of the Bailey III test) 3. GMS (gross motor skills scale)	1. FMSS, GMS between CG and GW under one year of age, *p* < 0.001 2. Over one year of age, GMS *p* = 0.02, FMSS *p* = 0.03 Significant statistical differences between the two groups
Erdogan Kavlak et al. [23]. Randomised controlled trial	*n* = 23 EG1: 12 (6/6) EG2: 11 (7/4)	EG1: Bobath therapy exercises EG2: Vojta therapy exercises	Both groups received 12 sessions, 2 days per week, for 6 weeks.	1. AIMS (Alberta Infant Mobility Scale) 2. BECK Depression Scale applied to mothers of patients 3. NHP (Nottingham Health Profile for measuring quality of life)	1. On the AIMS scale, both EG1 and EG2 had a statistical difference between initial and post-therapy measurements *p* = 0.001. There were no statistically significant differences between the two groups. However, no statistically significant differences were reported on the Beck and NHP scales.
Matteo Giuriato et al. [27]. Pilot study Pre/post therapy	*n* = 18 EG: 18 CG:0	EG: e-games exercises, via telerehabilitation	EG: 10 min warm-up with mobility exercises. 10 min of exercises to increase muscle strength. Circuit training with body weight. 10 min of exercises to improve body coordination. 10 min of stretching. 3 days /week/ 15 weeks	1. KTK (Kiphard and Schilling test for gross motor coordination and balance) 2. Systolic blood pressure 3. Weight 4. 6MWT (6 min walk test) 5. Standing long jump	In the KTK test, significant statistical differences were observed in backward walking (*p* = 0.002), systolic blood pressure (*p* = 0.04), and balance (*p* = 0.002). In the other parts of the test, weight or 6MWT, there were no statistically significant differences.
Myo Thein Tun et al. [22]. Pilot study Pre/post treatment	*n* = 5 (2/3) EG:5 CG:0	Structured group exercises to improve coordination and gross motor skills.	Group physical therapy exercises. 50 min/session 3 sessions/week 3 weeks	1. FMS (fine motor skills scale) 2. TGMD (gross motor development test) for static balance and lower limb strength. 3. Test of movements from sitting to standing 5 times	Significant statistical difference in the FMS test, *p* < 0.001, between pre- and post-therapy. The same applies to lower limb strength (*p* = 0.006), but not to static balance.

Note: “EG”—experimental group (received therapy) “CG”—control group

**Table 4:** STROBE methodological quality checklist.

Section Evaluated	Item	H. Towse et al. [24].	Feyzullah et al. [25].	Matteo G. et al. [27].	Myo Thein Tun et al. [22].
Title and abstract	1	✓	✓	✓	✓
I: context	2	✓	✓	✓	✓
I: objectives	3	✓	✓	✓	✓
M: study design	4		✓	✓	✓
M: context	5		✓	✓	✓
M: participants	6	✓	✓	✓	✓
M: results	7		✓	✓	✓
M: data sources/measures	8		✓	✓	✓
M: biases	9	✓			
M: sample size	10	✓	✓	✓	
M: quantitative variables	11	✓	✓	✓	✓
M: statistical methods	12		✓	✓	
R: participants	13	✓	✓	✓	✓
R: descriptive information	14	✓	✓	✓	✓
R: variable information results	15	✓	✓	✓	✓
R: main results	16	✓	✓	✓	✓
R: other analyses	17		✓	✓	
D: main results	18	✓	✓	✓	✓
D: limitations	19	✓	✓	✓	✓
D: interpretation	20	✓	✓	✓	✓
D: generalizability	21	✓		✓	
D: other information: funding	22	✓	✓	✓	

Note: “I”—Introduction; “M”—Materials; “R”—Results; “D”—Discussion

**Table 5:** Methodological quality assessment table on the PEDro scale.

Author, Year	Item 1	Item 2	Item 3	Item 4	Item 5	Item 6	Item 7	Item 8	Item 9	Item 10	Item 11	Total
Kavlak et al. [23]	**0**	**1**	**0**	**1**	**0**	**0**	**0**	**1**	**1**	**1**	**1**	**6/10**
Santos et al. [26]	**1**	**0**	**0**	**1**	**1**	**0**	**0**	**1**	**1**	**1**	**1**	**6/10**

Note: “1” indicates that a study meets the criterion, while “0” means that the study does not meet the criterion or does not provide sufficient information to confirm this. Item 1 = eligibility criteria were specified (not applicable to scoring articles on the PEDro scale); Item 2 = participants were randomly assigned to groups; Item 3 = treatment allocation was concealed; Item 4 = groups were similar at baseline; Item 5 = participants were blinded; Item 6 = therapists who administered the treatment were blinded; Item 7 = assessors who collected measurements were blinded; Item 8 = measurements for at least one primary outcome were obtained from more than 85% of participants initially assigned to groups; Item 9 = results for all participants who received treatment were reported in the control group, or when this was not possible, data for at least one primary outcome were analysed according to the “intention to treat” principle; Item 10 = statistical comparisons between groups were reported for at least one primary outcome; Item 11 = the study provides point estimates and measures of variability for at least one primary outcome [20].

## Data Availability

The data presented in this study are available on request from the first author due to ethical and data protection considerations.

## Abbreviations

The following abbreviations are used in this manuscript:

PR	Paediatric Rehabilitation
PT	Physical Therapy
DS	Down Syndrome

## Appendix A

### Appendix A.2. Strobe Statement

	**Item No**	**Recommendation**
**Title and abstract**	1	(*a*) Indicate the study’s design with a commonly used term in the title or the abstract.
		(*b*) Provide in the abstract an informative and balanced summary of what was done and what was found.
**Introduction**		
Background/rationale	2	Explain the scientific background and rationale for the investigation being reported.
Objectives	3	State-specific objectives, including any prespecified hypotheses.
**Methods**		
Study design	4	Present key elements of the study design early in the paper
Setting	5	Describe the setting, locations, and relevant dates, including periods of recruitment, exposure, follow-up, and data collection.
Participants	6	(*a*) Give the eligibility criteria, and the sources and methods of selection of participants.
Variables	7	Clearly define all outcomes, exposures, predictors, potential confounders, and effect modifiers. Give diagnostic criteria, if applicable.
Data sources/ measurement	8	For each variable of interest, give sources of data and details of methods of assessment (measurement). Describe comparability of assessment methods if there is more than one group.
Bias	9	Describe any efforts to address potential sources of bias.
Study size	10	Explain how the study size was arrived at.
Quantitative variables	11	Explain how quantitative variables were handled in the analyses. If applicable, describe which groupings were chosen and why.
Statistical methods	12	(*a*) Describe all statistical methods, including those used to control for confounding.
		(*b*) Describe any methods used to examine subgroups and interactions.
		(*c*) Explain how missing data were addressed.
		(*d*) If applicable, describe analytical methods taking account of sampling strategy.
		(*e*) Describe any sensitivity analyses.
**Results**		
Participants	13	(a) Report numbers of individuals at each stage of study—eg numbers potentially eligible, examined for eligibility, confirmed eligible, included in the study, completing follow-up, and analysed.
		(b) Give reasons for non-participation at each stage.
		(c) Consider use of a flow diagram.
Descriptive data	14	(a) Give characteristics of study participants (eg demographic, clinical, social) and information on exposures and potential confounders.
		(b) Indicate number of participants with missing data for each variable of interest.
Outcome data	15	Report numbers of outcome events or summary measures.
Main results	16	(*a*) Give unadjusted estimates and, if applicable, confounder-adjusted estimates and their precision (e.g., 95% confidence interval). Make clear which confounders were adjusted for and why they were included.
		(*b*) Report category boundaries when continuous variables were categorised.
		(*c*) If relevant, consider translating estimates of relative risk into absolute risk for a meaningful time period.
Other analyses	17	Report other analyses done—eg analyses of subgroups and interactions, and sensitivity analyses.
**Discussion**		
Key results	18	Summarise key results with reference to study objectives.
Limitations	19	Discuss limitations of the study, taking into account sources of potential bias or imprecision. Discuss both direction and magnitude of any potential bias.
Interpretation	20	Give a cautious overall interpretation of results considering objectives, limitations, multiplicity of analyses, results from similar studies, and other relevant evidence.
Generalisability	21	Discuss the generalisability (external validity) of the study results.
**Other information**		
Funding	22	Give the source of funding and the role of the funders for the present study and, if applicable, for the original study on which the present article is based.
